# Editorial: 2023 symposium on parathyroid fluorescence

**DOI:** 10.3389/fendo.2024.1434058

**Published:** 2024-06-20

**Authors:** Ilaria Di Meglio, Saba P. Balasubramanian, Richard Jaepyeong Cha, Quan-Yang Duh, Kerstin Lorenz, Anita Mahadevan-Jansen, Frédéric Triponez

**Affiliations:** ^1^ Department of Thoracic and Endocrine Surgery, University Hospitals of Geneva, Geneva, Switzerland; ^2^ Department of General Surgery, Sheffield Teaching Hospitals NHS Foundation Trust, Sheffield, United Kingdom; ^3^ Department of Pediatrics, George Washington University School of Medicine and Health Sciences, Washington, DC, United States; ^4^ Department of Surgery, University of California, San Francisco, San Francisco, CA, United States; ^5^ Department of Visceral, Vascular and Endocrine Surgery, Martin Luther University Halle-Wittenberg, Halle (Saale), Germany; ^6^ Department of Biomedical Engineering, Vanderbilt University, Nashville, TN, United States

**Keywords:** hypoparathyroidism, parathyroid, parathyroid fluorescence, thyroidectomy, parathyroidectomy

## Navigating the complexity of thyroid and parathyroid surgery—challenges and solutions

With the discovery of parathyroid autofluorescence (AF) nearly two decades ago (1), the field has witnessed exciting progress in research and innovative techniques for parathyroid gland (PG) identification and preservation. Nevertheless, amidst this exciting progress, persistent controversies have also arisen. This Research Topic aims to compile some of the latest insights and advancements in the field. Many articles featured were presented during the 5th Symposium on Parathyroid Fluorescence held in Geneva, Switzerland, in February 2023 organized by the International Society of Innovative Technologies for Endocrine Surgery (ISITES). This annual meeting aims to provide a collaborative platform for experts to address emerging challenges and solutions related to real-time PG identification during surgery. Together, these efforts aim to reduce the prevalence of post-surgical hypoparathyroidism (PoSH) and optimize clinical outcomes in parathyroid surgery.

## Addressing clinical and economic implications

Accurate identification of normal PGs during surgery is crucial but remains a significant challenge for surgeons. Failure to identify PGs can lead to inadvertent PG damage or excision, resulting in hypoparathyroidism and hypocalcaemia. PoSH represents the most frequent complication following thyroid surgery, with reported rates varying widely from 1% to 30% (2).

This variability in complication rates may be due to various reasons, including variations in outcome parameters (including their definition) and also the subjective nature of PG identification and its reliance on surgeon experience. It is worth noting that most studies are carried out in expert centres, potentially underestimating the true incidence of PoSH. In a recent study, Abood et al. addressed this issue by investigating postoperative hypoparathyroidism rates in a low-volume institution without parathyroid expertise, revealing a notable increase in rates. These findings underscore the need for future research in such settings.

While the clinical implications of postoperative hypoparathyroidism are well described, the literature lacks a quantification of its economic burden on healthcare systems. Addressing this gap, Benmiloud et al. examined the economic impact of postoperative hypoparathyroidism within the first year after total thyroidectomy for cancer in France. While their findings revealed a relatively modest yet significant additional cost to patients, this study underscores the need for long-term studies to comprehensively assess the economic burden of this condition.

## Beyond the naked eye: enhancing parathyroid preservation

Traditionally, surgeons have relied on naked-eye (NE) assessment to identify PGs during surgery. However, their small size and resemblance to surrounding tissue make visual identification of PGs a daunting task.

While the consensus among surgeons leans towards identifying PGs to reduce inadvertent PG damage and subsequent complications, some surgeons argue otherwise. In a mini-review, Llorente-Poch et al. summarized existing controversies, highlighting that most studies opposing PG identification suffer from inherent limitations in study design. These limitations include retrospective design, conservative surgical procedures, and small sample sizes. While advocating for PG identification to prevent inadvertent excision and hypoparathyroidism, Llorente-Poch et al. also draw attention to the shift from visual identification towards more promising alternatives like near-infrared autofluorescence (NIRAF) imaging and indocyanine green (ICG) angiography.

The discovery of parathyroid AF has sparked significant enthusiasm among surgeons and researchers alike, driving extensive research on NIRAF imaging as an intraoperative adjunct for PG localization (3, 4). This unique property of PGs enables real-time and non-invasive PG localization, offering potential benefits in reducing the incidence of PoSH.

Though NIRAF cannot assess PG vascularity and thus viability, it can be combined with intravenous injection of ICG during surgery. This combined approach enables PG visualization while safeguarding blood supply and perfusion. Moreno-Llorente et al. introduced a study protocol for evaluating the efficacy of ICG angiography-guided thyroidectomy in preventing postoperative hypoparathyroidism. Their randomized, single-blind, controlled, parallel-arm, multicentre trial aims to compare the rate of permanent hypoparathyroidism between ICG angiography and conventional thyroidectomy, with anticipated insights into its benefits. Similarly, Vabalayte et al. also highlighted the efficiency of intraoperative ICG angiography and a less characterized dye, Brilliant Green, highlighting improved outcomes compared to the NE assessment.

## Distinguishing healthy from diseased PGs

Accurate identification of parathyroid adenomas also presents a key challenge in surgery for primary or renal hyperparathyroidism (5). Reported variability in parathyroid autofluorescence suggests that NIRAF imaging could aid in detecting abnormal glands (6, 7) and improve cure rates. However, the impact of NIRAF on cure rates remains inconclusive. For instance, Pannu et al. found no improvements in cure rates with NIRAF in their study on first-time surgery for primary hyperparathyroidism (PHPT). This lack of improvement may stem from the low power of this study, the relatively low sensitivity of the current technology, and the potential for false positives. There is also limited understanding of the underlying reasons for differences in diseased PG intensities. Huang used the commercially available Fluobeam system to address this issue, revealing that autofluorescence depends on factors like tissue content and capsule thickness, shedding light on why not all studies report these intensity differences (8, 9).

An alternative approach that may improve success rates and reduce the need for extensive dissection is intraoperative parathyroid hormone (PTH) (IOPTH) monitoring, which detects significant falls in PTH levels. Kumar et al. conducted a pilot study comparing a traditional lab-based IOPTH assay to the Novel Biomarkers Catalyst Lab (NBCL) CONNECT assay, which enables intraoperative PTH assessment within minutes. While the NBCL CONNECT assay shows promise in reducing theatre times, concerns about false negatives prompt further refinement.

## Exploring novel methodologies

Despite variability among studies, NIRAF devices hold promise as a potential standard practice among surgeons, with ongoing efforts to refine NIRAF devices and explore novel prototypes. One example of this endeavour is the recently developed hANDY-I prototype, which delivers simultaneous anatomical localization and fluorescence visualization of PGs.

In an early feasibility study focused on hANDY-I, Ali et al. demonstrated its utility for the real-time identification of PGs during surgery. Their findings reveal that out of 75 PGs inspected, hANDY-I could reliably detect 71 PGs showing strong autofluorescence signals. Similarly, Seo et al. explored the feasibility of hANDY-I in detecting PGs via the transoral endoscopic thyroidectomy vestibular approach (TOETVA), a scarless and remote-access thyroidectomy technique. While large-scale clinical trials are essential to validate these findings, these studies underscore the promising role of this, in both standard thyroidectomy and TOETVA, offering potential enhancements to patient safety.

Using a different lab-built NIRAF imaging system, Han et al. investigated an unexplored property of NIRAF devices: the depth range at which previously unexposed PGs can be detected during thyroidectomy. Their findings revealed a maximum depth of 3.05 mm and an average depth of 1.23 ± 0.73 (mean ± SD). While these depths may vary in commercial systems, a better understanding of the detection depth may help mitigate challenges faced by novice surgeons.

Other studies also explored alternative models for *in vitro* and *in vivo* studies, offering new avenues of research in this field. In their study, Sekhar et al. used patient-derived parathyroid organoids to explore parathyroid metabolism, shedding light on parathyroid-related diseases like PHPT.

## Conclusions

Evaluating the current body of literature on NIRAF and ICG reveals notable variability in study designs and ambiguity regarding their efficacy in reducing postoperative complications. However, most published studies report better parathyroid detection rate using NIRAF techniques and less overall short- and medium-term PoSH (10). More studies are necessary to demonstrate the impact on long-term PoSH. Regardless, NIRAF imaging systems offer a promising non-invasive approach that leverages the intrinsic AF of PGs to facilitate real-time PG localization during surgery. When employed in conjunction with fluorophores such as ICG, parathyroid viability can also be assessed. As underscored by Yuan et al. in their comprehensive review addressing both challenges and future prospects, despite the undeniable heterogeneity in accuracy and precision, these technologies have the potential to become invaluable tools for enhancing surgical decision-making.

## Author contributions

ID: Writing – original draft, Writing – review & editing. SB: Writing – review & editing. RC: Writing – review & editing. KL: Writing – review & editing. QD: Writing – review & editing. AM: Writing – review & editing. FT: Writing – original draft, Writing – review & editing.
